# Evaluation of Thermal Aging Susceptibility of Recycled Waste Plastic Aggregates (Low-Density Polyethylene, High-Density Polyethylene, and Polypropylene) in Recycled Asphalt Pavement Mixtures

**DOI:** 10.3390/polym17060731

**Published:** 2025-03-10

**Authors:** Yeong-Min Kim, Kyungnam Kim

**Affiliations:** 1 Department of Highway & Transportation Research, Korea Institute of Civil Engineering and Building Technology, 283 Goyangdae-ro, Ilsanseo-gu, Goyang-si 10223, Gyeonggi-do, Republic of Korea; 2 Pavement Research Division, Korea Expressway Corporation Research Institute, Dong-tansunhwan-daero 17-gil, Hwaseong-si 18489, Gyeonggi-do, Republic of Korea

**Keywords:** crushed recycled marble stone powder, SBS polymer, recycled tire rubber, sustainable asphalt, innovative filler, environmental impact, dynamic modulus

## Abstract

The increasing demand for sustainable road construction materials necessitates innovative solutions to overcome the challenges of Recycled Asphalt Pavement (RAP), including aged binder brittleness, reduced flexibility, and durability concerns. Waste Plastic Aggregates (WPA) offer a promising alternative; however, their thermal aging behavior and interactions with RAP remain insufficiently understood. This study evaluates the performance of RAP-based asphalt mixtures, incorporating three types of WPA—Low-Density Polyethylene (LDPE), High-Density Polyethylene (HDPE), and Polypropylene (PP)—under three thermal aging conditions: mild (60 °C for 7 days), moderate (80 °C for 14 days), and severe (100 °C for 30 days). The mixtures were designed with 30% RAP content, 10% and 20% WPA by aggregate weight, and SBS-modified binder rejuvenated with 2% and 4% sewage sludge bio-oil by binder weight. It is considered that thermal aging may impact the performance of WPA in RAP mixtures; therefore, this study evaluates the durability and mechanical properties of RAP mixtures incorporating LDPE, HDPE, and PP under varying thermal aging conditions to address these challenges. The results showed that incorporating WPA and bio-oil significantly enhanced the mechanical performance, durability, and sustainability of asphalt mixtures. Marshall Stability increased by 12–23%, with values ranging from 12.6 to 13.2 kN for WPA-enhanced mixtures compared to 12.7 kN for the control. ITS improved by 15–20% in dry conditions (1.34–1.44 MPa) and 12–18% in wet conditions (1.15–1.19 MPa), with TSR values reaching up to 82.64%. Fatigue life was extended by 28–43%, with load cycles increasing from 295,600 for the control to 352,310 for PP mixtures. High-temperature performance showed a 12–18% improvement in softening point (57.3 °C to 61.2 °C) and a 23% increase in rutting resistance, with rut depths decreasing from 7.1 mm for the control to 5.45 mm for PP mixtures after 20,000 passes. These results demonstrate that combining RAP, WPA, and bio-oil produces sustainable asphalt mixtures with superior performance under aging and environmental stressors, offering robust solutions for high-demand applications in modern infrastructure.

## 1. Introduction

RAP has emerged as a sustainable alternative in road construction, aiming to reduce the consumption of virgin materials and lower environmental impacts. The utilization of RAP in asphalt mixtures aligns with global initiatives promoting resource conservation and circular economy practices [1]. However, despite its potential, the integration of RAP poses significant challenges, primarily associated with the aged binder present in recycled materials [2]. This aged binder is often stiffer and more brittle, leading to reduced flexibility and durability of asphalt pavements. These shortcomings are particularly pronounced under extreme conditions, such as high traffic loads, elevated temperatures, or moisture exposure [3].

The utilization of RAP in asphalt mixtures has been extensively studied for its potential to reduce environmental impacts and promote sustainable construction practices [4]. Xue et al. emphasized that incorporating RAP reduces the demand for virgin aggregates and asphalt binders [5], lowering greenhouse gas emissions and raw material costs [6]. Innovative warm mix asphalt (WMA) technologies now allow RAP to be processed at lower temperatures, reducing energy consumption and greenhouse gas emissions [7,8,9]. Additives such as rejuvenators, including bio-based and polymer-modified compounds, restore the aged binder in RAP, enhancing its flexibility and long-term performance [10,11,12,13,14]. Advanced milling and crushing techniques ensure optimal particle size distribution and adhesion in the recycled mix [15]. Additionally, technologies like foamed asphalt and high RAP-content designs enable higher incorporation rates of recycled materials without compromising pavement durability [15]. Digital technologies, including real-time quality control systems and performance modeling software, further optimize RAP blending and application. These advancements collectively make recycled asphalt a viable solution for sustainable infrastructure development [16].

However, Wang et al. found that the aged binder in RAP often results in increased stiffness and brittleness, which can compromise the flexibility and durability of asphalt mixtures under heavy traffic loads [17]. To address these limitations, researchers have explored various rejuvenation methods. Ali et al. demonstrated that bio-oils derived from waste biomass can rejuvenate aged binders, improving ductility and extending fatigue life in RAP-based mixtures [18]. Similarly, Xue et al. highlighted that bio-oil, due to its high aromatic content, effectively restores the adhesive and viscoelastic properties of aged binders [6]. The environmental benefits of bio-oils, such as diverting waste from landfills and supporting a circular economy, have also been emphasized by Lee et al [19].

Recent studies have also explored the incorporation of waste plastics in asphalt mixtures to develop eco-friendly and performance-enhanced pavements. For instance, research by Agha et al. [20] evaluated the performance of Hot Mix Asphalt using polyethylene terephthalate (PET) with wet and dry mixing techniques, demonstrating improvements in mechanical properties and sustainability. Similarly, Lee et al. investigated asphalt mixtures containing waste plastic aggregates and additives like magnesium and fly ash, highlighting their potential for reducing environmental impact [12]. Xiao et al. [21] examined the thermodynamic properties of aggregate coated with different plastic types, focusing on adhesion and moisture resistance, while Ullah et al. [22] characterized asphalt concrete with low- and high-density polyethylene as aggregates. Other studies, such as Audy et al. [23], employed a multi-criteria screening tool to optimize recycled plastic selection for sustainable asphalt. Innovative applications, like plastic waste hydrophobic coatings (Xiao et al. [24]) and blends with crumb rubber (Prathibha and Karthik [25]), further emphasize the versatility of waste plastics in enhancing asphalt performance. Collectively, these studies underscore the viability of using waste plastics in asphalt to promote durability, sustainability, and noise reduction in pavements.

The increasing global emphasis on sustainable construction practices has highlighted the potential of RAP as a viable alternative to virgin asphalt in road construction. While the use of RAP offers significant environmental benefits, such as reduced reliance on natural aggregates and decreased carbon emissions, its widespread adoption is hindered by several critical challenges. A primary concern is the stiff and brittle nature of the aged binder in RAP, which compromises the flexibility, fatigue resistance, and overall durability of asphalt mixtures, particularly under high traffic loads and extreme weather conditions.

To mitigate these challenges, additives like WPA have been introduced to enhance the mechanical properties of asphalt mixtures. WPA not only promotes the recycling of plastic waste but also improves mixture stability, deformation resistance, and longevity. However, WPA materials are prone to aging and thermal degradation, which can adversely affect their performance in asphalt pavements. Furthermore, different types of plastics—such as LDPE, HDPE, and PP—exhibit varied responses to thermal aging, yet their specific behaviors and impacts under different aging conditions remain insufficiently understood. Another critical limitation lies in the lack of comprehensive studies that address the combined use of RAP and WPA under varying thermal aging scenarios. While existing research has explored the benefits of using RAP and WPA independently, the interactions between these components under real-world conditions have yet to be fully investigated. Questions remain about the durability, long-term performance, and optimal mix design of asphalt pavements that integrate RAP, WPA, and rejuvenators like bio-oils.

This study systematically examines the performance of RAP-based asphalt mixtures incorporating different types of WPA under multiple thermal aging conditions. It aims to optimize the use of RAP and WPA to enhance mechanical properties, durability, and sustainability in asphalt pavements. The novelty of this research lies in evaluating the combined effects of RAP, WPA, and sewage sludge bio-oil as a rejuvenator under controlled thermal aging conditions, providing comprehensive insights into their long-term performance. This work contributes to the development of high-performing, environmentally sustainable asphalt mixtures suitable for diverse climates and heavy traffic conditions. The mixtures incorporate three types of WPA—LDPE, HDPE, and PP—and are subjected to three thermal aging levels: mild (60 °C for 7 days), moderate (80 °C for 14 days), and severe (100 °C for 30 days). A fixed RAP content of 30% is combined with virgin aggregates, SBS-modified binder, and WPA at 10% and 20% by weight of the aggregate. Sewage sludge bio-oil, used as a rejuvenator, is added at 2% and 4% by weight of the binder. Performance evaluation includes tests such as Marshall Stability, ITS, Fatigue Life, Dynamic Modulus, and Hamburg Wheel Tracking. These tests are conducted under realistic conditions to simulate tropical climates and heavy traffic. This study offers insights into material interactions and contributes to the development of durable and sustainable asphalt mixtures.

## 2. Materials

### 2.1. Overview About the Materials

The materials utilized in this study were chosen to evaluate the impact of thermal aging on WPA and its integration into RAP. The primary focus was to ensure compatibility, sustainability, and performance under varying conditions. RAP was sourced from milled asphalt pavements and carefully selected to maintain consistent quality. RAP was included at 30% by weight of the total mixture, a proportion that balances environmental benefits with performance considerations. The aged binder within the RAP contributes stiffness to the mixture, which requires rejuvenation to restore flexibility. Prior to mixing, the properties of the RAP, such as residual binder content and aggregate gradation, were assessed to ensure its suitability for the experimental designs.

Three types of WPA—LDPE, HDPE, and PP—were incorporated to study their individual and collective responses to thermal aging. Each WPA type was sourced from post-consumer plastic waste and processed into aggregate sizes ranging from 5 mm to 13 mm. The WPA content was fixed at 10% by weight of the total aggregate to investigate its performance while maintaining the structural integrity of the asphalt mixture. An SBS-modified binder was used as the primary asphalt binder due to its superior performance in enhancing elasticity, deformation resistance, and overall durability. To rejuvenate the aged binder in RAP, sewage sludge bio-oil was selected for its ability to restore binder flexibility and improve compatibility with WPA. This bio-oil, produced via the pyrolysis of municipal sewage sludge, aligns with the study’s focus on sustainability by repurposing waste materials. The gradation of aggregates, including virgin and recycled materials, was optimized to ensure structural stability and performance. Virgin aggregates were sourced from local quarries and blended with RAP and WPA to achieve a well-graded mixture. This approach ensured that the experimental designs addressed both mechanical performance and environmental considerations effectively.

### 2.2. Aggregates

#### 2.2.1. Natural Aggregate

The natural aggregates used in this study were sourced from local quarries, consisting primarily of crushed granite. These aggregates were selected for their excellent mechanical properties, including high strength and durability. They were processed to meet the required gradation for asphalt mixtures, ensuring structural integrity and resistance to deformation. The specific gravity and water absorption properties of the natural aggregates were optimized to align with the standard specifications for base and surface layers.

#### 2.2.2. RAP Aggregate

RAP aggregates were obtained from previously milled asphalt pavements. These aggregates retained residual aged binder, contributing to the sustainability of the mixture by reducing the need for virgin materials. The RAP aggregates exhibited slightly lower specific gravity compared to natural aggregates, reflecting the presence of aged binder and finer particles. Properties such as gradation and binder content were evaluated to ensure compatibility with the new mixture designs.

It should be noted that the lower specific gravity of RAP aggregates compared to natural aggregates influences the overall density, compaction characteristics, and mechanical performance of RAP-based mixtures. This reduction in density can lead to higher air void content, affecting workability and durability. Additionally, aged binder coatings on RAP particles may hinder proper compaction, necessitating the use of rejuvenators such as bio-oil to enhance binder–aggregate cohesion and mitigate potential performance drawbacks. Studies on recycled aggregate concrete [26,27] have similarly highlighted the impact of lower specific gravity on mechanical behavior, emphasizing the need for optimized mix designs to ensure long-term performance. The incorporation of findings from recycled aggregate concrete research into asphalt mixtures provides valuable insights into improving sustainability and mechanical properties in pavement applications.

#### 2.2.3. WPA

**Figure 1:**
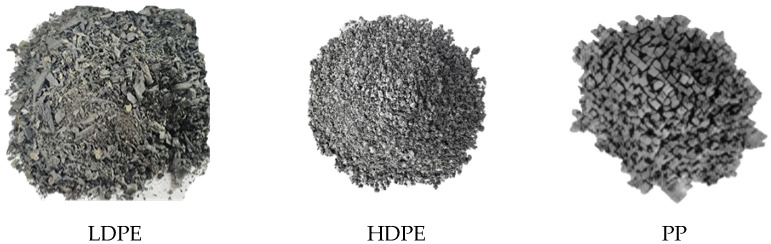
WPA used in this research.

WPA was processed from post-consumer plastic waste, including LDPE, HDPE, and PP (see Figure 1 and Table 1). The WPA particles were sized between 5 mm and 13 mm and were designed to partially replace natural aggregates. Due to their lower density, WPA contributes to a lighter mixture and improves flexibility. The thermal and mechanical properties of WPA were characterized to evaluate its compatibility and performance in asphalt mixtures under thermal aging.

The production of WPA in this study was carried out using a controlled and standardized process to ensure uniformity and quality. Post-consumer plastic waste, including LDPE, HDPE, and PP, was sourced from municipal recycling facilities. These materials were thoroughly cleaned to remove contaminants such as dirt, oil, and adhesives. The cleaning process involved washing the plastics with a mild detergent solution, followed by rinsing with deionized water and air-drying.

Once cleaned, the plastics were shredded into smaller pieces using an industrial shredder. The shredded plastic was then subjected to a thermal process to convert it into aggregate-sized particles. This process involved heating the plastic in an extrusion machine at temperatures ranging from 160 °C to 180 °C, depending on the plastic type. For LDPE, the temperature was maintained at the lower range (160–165 °C), while HDPE and PP required higher temperatures (170–180 °C) due to their higher melting points.

The molten plastic was extruded through a die plate, forming long strands, which were then cooled rapidly using a water bath to prevent thermal degradation. These strands were chopped into aggregate-sized particles ranging from 5 mm to 13 mm in diameter using a granulator. The particle size distribution was carefully monitored to ensure compatibility with the gradation requirements of asphalt mixtures.

To enhance the mechanical properties of WPA, additives such as fly ash and steel slag were incorporated during the extrusion process (see Table 2). Fly ash contributed to improved stiffness and binding properties, while steel slag provided additional strength and durability. The mixture of plastic and additives was homogenized in the extrusion chamber to ensure even distribution.

The final WPA particles were subjected to quality control tests to verify their specific gravity, water absorption, and thermal stability. Specific gravity was measured using ASTM C127 [28], while water absorption was determined using ASTM C128 [29]. A Thermogravimetric Analysis (TGA) was conducted to assess the thermal stability of the aggregates, ensuring they could withstand the temperatures encountered during asphalt production without degradation.

**Figure 2:**
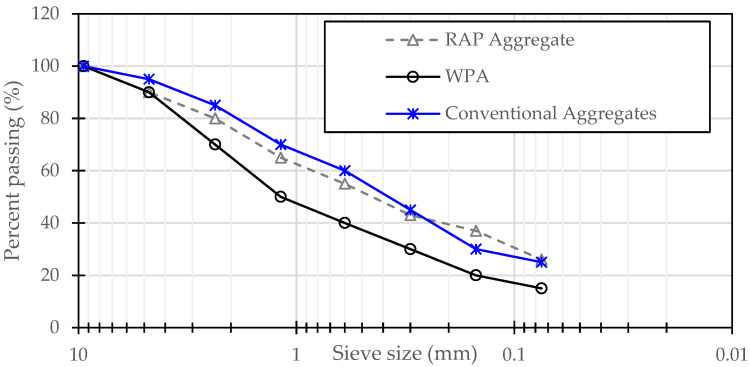
Gradation of material used in this research.

The resulting WPA was stored in moisture-free conditions to maintain its quality before being incorporated into asphalt mixtures. Table 1 summarizes the production parameters and key properties of WPA produced in this study and Figure 2 presents the aggregate gradations.

The WPA content in this study was selected at 10% and 20% by aggregate weight based on prior research and preliminary trials, which indicated that these levels provide an optimal balance between mechanical performance, durability, and workability. While increasing WPA content beyond 20% may further enhance specific properties, such as stiffness or rutting resistance, it could also introduce challenges related to excessive mixture stiffness, workability issues, and potential cracking. Future research will focus on evaluating the feasibility of higher WPA concentrations (e.g., 25% or 30%) and their effects on mechanical performance and long-term durability under various environmental and loading conditions.

### 2.3. Asphalt Binder

The asphalt binder used in this study was an SBS-modified bitumen, selected for its superior performance in enhancing the elasticity, deformation resistance, and overall durability of asphalt mixtures. This type of binder is particularly well-suited for high-stress environments and tropical climates, where high temperatures and heavy traffic loads can accelerate binder aging and pavement distress.

The SBS-modified binder was characterized by its penetration grade, softening point, and dynamic shear modulus. The penetration grade of the binder was 70 dmm, indicating moderate stiffness, which is essential for maintaining flexibility under varying conditions. The softening point was measured at 65 °C, ensuring that the binder could withstand elevated temperatures without becoming excessively soft. The dynamic shear modulus (G*/sin δ) was 1.25 kPa at 64 °C, reflecting its ability to resist permanent deformation under shear stress.

In this study, sewage sludge bio-oil was introduced as a rejuvenator to restore the properties of the aged binder in the RAP. The bio-oil was blended with the SBS-modified binder using the wet method, where it was incorporated at varying concentrations of 2% and 4% by weight of the binder. The bio-oil served to reduce the stiffness of the aged binder, improve workability, and enhance the overall flexibility of the mixture.

To further improve moisture resistance, a commercial antistripping agent (Evotherm) was added at 0.5% by weight of the total binder content. This additive was selected for its proven ability to enhance adhesion between the binder and aggregates, reducing the risk of moisture-induced damage such as stripping or raveling.

The properties of the SBS-modified binder and its blends with bio-oil were evaluated to ensure their compatibility with the experimental mix designs. Table 2 summarizes the key properties of the asphalt binder used in this study.

The SBS-modified asphalt binder exhibits several key properties that enhance its performance. It has a penetration value of 70 dmm [30], a softening point of 65 °C [31], and a ductility of 120 cm [32], indicating good flexibility and resistance to cracking. The binder demonstrates excellent rheological characteristics with a dynamic shear modulus of 1.25 kPa [33] and an elastic recovery of 75% [34], which contributes to its ability to resist deformation under stress. It has a high flash point of 250 °C [35], signifying good thermal stability, and its rotational viscosity at 135 °C is 3.0 Pa·s [36], indicating manageable workability. Additionally, the binder shows minimal separation, with a result of only 2% [37].

## 3. Methods

### 3.1. Mix Design

The mix design process was conducted to develop asphalt mixtures that integrate three types of WPA with RAP at varying thermal aging levels. The primary objective was to optimize the performance, durability, and sustainability of these mixtures, ensuring their suitability for tropical climates and heavy traffic conditions. To simulate the effects of environmental exposure over time, the WPA underwent controlled thermal aging at three levels. Level 1, representing mild aging, involved subjecting the WPA to 60 °C for seven days, simulating short-term exposure to moderate temperatures. Level 2, indicating moderate aging, exposed the WPA to 80 °C for 14 days, replicating prolonged exposure to elevated temperatures typical of storage or pre-use conditions. Level 3, corresponding to severe aging, involved subjecting the WPA to 100 °C for 30 days, simulating extended exposure to extreme heat, as might occur during production or in-service use in hot climates. This tiered aging protocol allowed for a comprehensive assessment of how thermal degradation affects the mechanical and thermal properties of WPA and its subsequent influence on the performance of asphalt mixtures.

#### 3.1.1. Design Methodology

The mix design followed the Marshall Method as per AASHTO T245 standards [38], with adjustments to accommodate the inclusion of RAP and WPA. The Optimum Asphalt Content (OAC) was determined for each mix by evaluating key properties such as Marshall Stability, flow, air voids (Va), and voids in mineral aggregate (VMA). The inclusion of sewage sludge bio-oil as a rejuvenator ensured the restoration of aged binder properties, improving flexibility and cohesion. The design incorporated the following factors:

- A fixed RAP content of 30% by weight of the total mixture.
- WPA content fixed at 10% by weight of the total aggregate.
- Three types of WPA (LDPE, HDPE, PP) subjected to three thermal aging levels (Level 1, Level 2, Level 3).

#### 3.1.2. Final Mix Design

The final mix design comprised a series of control and experimental mixtures. Table 3 summarizes the mix design parameters for each combination of RAP, WPA type, and thermal aging level.

### 3.2. Production Process

The mixing process for the asphalt mixtures was carefully designed to ensure uniform distribution of RAP, WPA, and rejuvenator within the asphalt binder. The process was optimized based on the thermal properties and aging behavior of the WPA types (LDPE, HDPE, and PP) to achieve a consistent and high-performing mix. The general mixing procedure involved heating the RAP and virgin aggregates, preheating the WPA, and blending these materials with the asphalt binder and sewage sludge bio-oil. The mixing process for each WPA type was adjusted to account for differences in melting temperatures, thermal stability, and compatibility with the binder. Table 4 presents the mixing process details for each WPA type.

The mixing process was tailored to the unique properties of each type of WPA to ensure optimal performance and uniformity in the asphalt mixtures. For LDPE, preheating was performed at 140 °C to soften the material without causing thermal degradation. During mixing, the temperature was maintained between 150 °C and 160 °C, allowing for the uniform distribution of LDPE particles within the mixture. Due to its lower melting temperature and faster diffusion characteristics, a shorter diffusion time of 30 min was sufficient for LDPE to interact effectively with the binder.

HDPE required higher preheating temperatures of 150 °C due to its higher melting point. To achieve the proper coating of HDPE particles with the binder, the mixing process was extended slightly, lasting 3–4 min. A diffusion time of 40 min was employed, providing adequate interaction between the aged binder and HDPE to enhance the mixture’s cohesiveness.

For PP, which has the highest melting point among the three WPA types, preheating was conducted at 160 °C. The mixing temperature range was maintained between 160 °C and 170 °C, ensuring thorough blending while avoiding temperatures that could exceed the binder’s flash point. Due to PP’s slower interaction with the binder, the diffusion time was extended to 50 min, allowing sufficient time for the material to integrate fully into the mixture.

General considerations were applied across all WPA types to maintain consistency and quality. WPA was added in solid form during mixing to prevent premature softening during preheating. The bio-oil rejuvenator was introduced gradually during the mixing process to ensure the uniform distribution and effective restoration of the aged RAP binder. Mixing times were carefully adjusted to achieve a proper coating of the aggregates without overheating, which could degrade the binder or WPA. Additionally, the post-mixing holding temperature was maintained consistently to prevent premature cooling during sample preparation, ensuring that the mixture retained its desired properties throughout the process.

### 3.3. Testing Method

To evaluate the performance of the asphalt mixtures containing WPA and RAP, a comprehensive series of laboratory tests was conducted. These tests assessed mechanical properties, durability, and resistance to environmental factors to ensure the mixtures met the stringent requirements for use in tropical climates. Each test was conducted on multiple specimens (typically three replicates per mixture), and the standard deviation was calculated for each data point. The testing program included the following methods:

#### 3.3.1. Marshall Stability and Flow Test

The Marshall Stability and Flow Test, conducted in accordance with ASTM D6927 [39], was used to measure the load-bearing capacity and deformation characteristics of the mixtures. Cylindrical specimens, with a diameter of 101.6 mm and a height of approximately 63.5 mm, were prepared using 75 blows per side of a standard Marshall compactor. Stability (maximum load the specimen can withstand) and flow (deformation under load) values were recorded at 60 °C. These metrics provided insights into the mixtures’ resistance to permanent deformation under high temperatures and heavy loads.

#### 3.3.2. ITS and Tensile Strength Ratio (TSR)

The ITS and TSR tests, following AASHTO T283 standards [40], evaluated the tensile strength and moisture susceptibility of the mixtures. The specimens were conditioned at 25 °C and tested under dry and wet conditions. The ITS values were measured by applying a compressive load along the diametrical plane until failure. TSR, calculated as the ratio of wet ITS to dry ITS, was used to assess the mixture’s resistance to moisture-induced damage.

#### 3.3.3. Fatigue Life Test

Fatigue life testing, performed as per AASHTO T321 [41], measured the resistance of the mixtures to cracking under repeated loading. Beam specimens (380 mm × 63 mm × 50 mm) were subjected to cyclic loading at a frequency of 10 Hz and a strain level of 0.5%. The number of load cycles to failure, defined as a 50% reduction in stiffness, indicated the durability of the mixtures under traffic-induced fatigue conditions.

#### 3.3.4. High-Temperature Performance Test

The high-temperature performance of the binder was assessed using the Dynamic Shear Rheometer (DSR) test as per ASTM D7175 [33]. Samples of the binder, extracted from the mixtures, were tested at temperatures of 64 °C and 70 °C to evaluate stiffness (G*/sin δ) and elastic recovery. Additionally, the softening point of the binder was determined using ASTM D36 to measure its resistance to deformation at elevated temperatures.

#### 3.3.5. Dynamic Modulus Test

**Figure 3:**
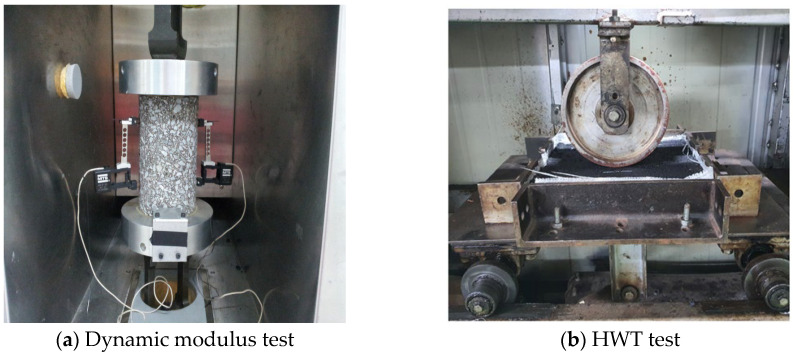
(**a**) Dynamic modulus test and (**b**) HWT test.

The stiffness of the mixtures under varying temperatures and loading frequencies was evaluated through the dynamic modulus test (see Figure 3a), following AASHTO T342 [42]. Cylindrical specimens (100 mm × 150 mm) were subjected to stress at five temperature settings (−10 °C to 54 °C) and loading frequencies (0.1 Hz to 25 Hz). The dynamic modulus (|E*|) values provided insights into the mixture’s ability to resist deformation under different traffic and environmental conditions.

#### 3.3.6. Hamburg Wheel Tracking (HWT) Test

Rutting resistance was assessed using the HWT Test as per AASHTO T324 [43]. Slab specimens (320 mm × 260 mm × 40 mm) were submerged in a water bath at 50 °C and subjected to repeated passes of a steel wheel applying a 700 N load. Rut depth measurements were taken at intervals to evaluate the mixture’s resistance to permanent deformation and moisture damage (see Figure 3b).

### 3.4. Lifecycle Cost Analysis (LCCA)

The LCCA method was utilized to evaluate the environmental and economic impacts of asphalt mixtures containing WPA and sewage sludge bio-oil, assessing the entire lifecycle of the pavement, from material production to end-of-life disposal. By incorporating simulated data based on prior research and expert recommendations, the study quantified environmental metrics such as CO_2_ emissions (kg/ton) from production and transportation, waste diversion (kg/ton) achieved through recycled materials, and recycled content (%). Economic metrics included raw material costs (USD/ton) covering RAP, WPA, and bio-oil, production costs (USD/ton) encompassing energy and labor for processes like WPA pelletization and bio-oil production, and lifecycle costs (USD/m^2^/year), reflecting maintenance and repair over the pavement’s lifespan. Scenarios were modeled for control mixtures and WPA-enhanced mixtures using LDPE, HDPE, and PP at varying aging levels, ensuring a comprehensive and realistic assessment of performance, cost-effectiveness, and sustainability. The simulated data inputs, drawn from the literature and expert recommendations, are summarized in Table 5.

The CO_2_ emissions were estimated using a LCA approach [44,45], incorporating the energy required for material production, transportation, and processing. Emission factors (kg CO_2_ per MJ) were applied to calculate the total CO_2_ emissions based on the energy consumed during RAP processing, WPA pelletization, bio-oil production, and asphalt mixing. The results were expressed in mass equivalent (kg CO_2_ per ton of asphalt mixture) to standardize comparisons. The LCA methodology ensures a comprehensive assessment of the environmental impact of each mixture, providing a more accurate estimation of CO_2_ reduction potential when incorporating WPA and RAP.

## 4. Results and Discussions

### 4.1. Marshall Stability and Flow Tests

**Figure 4:**
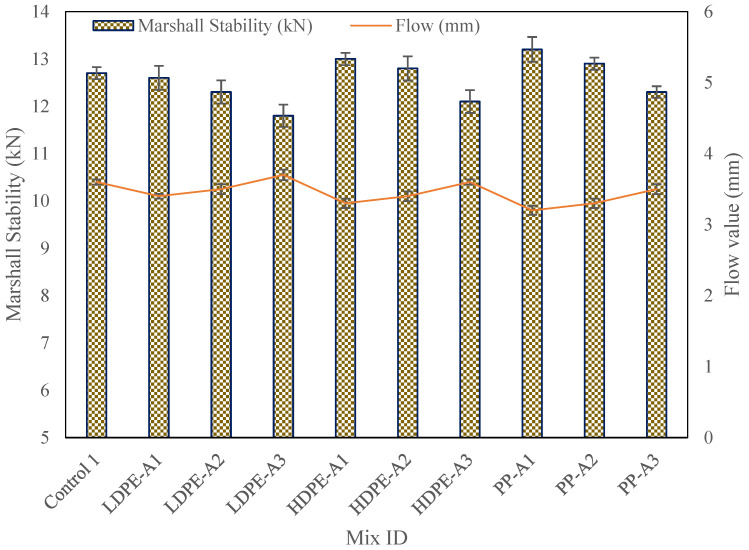
Marshall Stability test results.

The Marshall Stability and Flow Test results indicate that WPA incorporation significantly enhances asphalt mixture performance, with variations based on WPA type and aging level (Table 6, Figure 4). The control mixture (12.7 kN, 3.6 mm flow) exhibited lower stability and higher deformation compared to WPA-modified mixtures. Among WPA types, PP mixtures showed the highest stability (13.2 kN, 15% increase) and lowest flow (3.2 mm, 11% reduction), demonstrating superior load-bearing capacity and resistance to deformation. HDPE followed closely, while LDPE exhibited moderate gains but declined at higher aging levels.

Thermal aging increased mixture stiffness but also reduced flexibility, with LDPE-Aging3 showing the greatest decline (stability: 11.8 kN, flow: 3.7 mm). PP exhibited the best resistance to aging-induced degradation. The trends align with the mechanical properties of WPA, where PP’s higher rigidity provides stability, while LDPE’s susceptibility to thermal degradation affects performance. These findings underscore the need for optimized WPA selection to balance stability and durability under aging conditions.

In summary, the inclusion of WPA significantly improves the Marshall Stability and Flow characteristics of RAP-based asphalt mixtures, with PP showing the most promising results, particularly at lower aging levels. However, as thermal aging increases, the performance of all mixtures declines, highlighting the need for further optimization of WPA processing and integration methods to mitigate the effects of aging.

### 4.2. ITS and TSR

**Figure 5:**
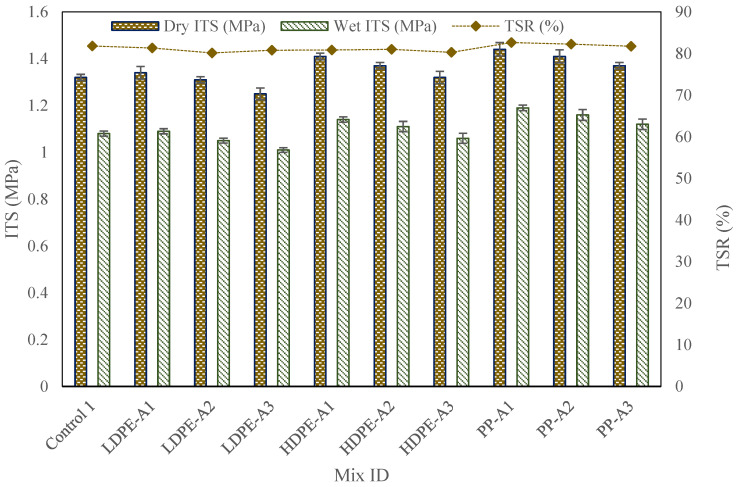
IST test results.

The precise results from the ITS and TSR tests reveal distinct performance differences across the mixtures, based on WPA type and thermal aging level (see Figure 5). The control mixture (Control 1), without any WPA or bio-oil, recorded the lowest dry ITS (1.32 MPa) and wet ITS (1.15 MPa), leading to a TSR of 81.82%. This underscores the control’s limited resistance to moisture-induced damage and overall moderate tensile performance.

The incorporation of WPA significantly improved both dry and wet ITS values, with PP-modified mixtures demonstrating the highest performance. PP-Aging1 exhibited the highest dry ITS (1.44 MPa) and wet ITS (1.19 MPa), achieving a TSR of 82.64%. Compared to the control mixture (1.32 MPa dry ITS, 1.08 MPa wet ITS, and 81.82% TSR), PP-Aging1 showed a 9.09% improvement in dry ITS, a 10.19% increase in wet ITS, and a 1.0% enhancement in TSR.

HDPE mixtures also performed well, with HDPE-Aging1 recording a dry ITS of 1.41 MPa and a TSR of 80.85%, indicating improved tensile strength and moisture resistance. LDPE mixtures exhibited moderate improvements, with LDPE-Aging1 achieving a dry ITS of 1.34 MPa and a TSR of 81.34%, showing slight gains over the control but lower performance compared to PP and HDPE. The superior performance of PP can be attributed to its higher rigidity and stronger binder interaction, which enhances cohesion and resistance to tensile forces. HDPE’s intermediate performance reflects its balance between stiffness and flexibility, while LDPE’s lower values may be due to its softer nature and higher susceptibility to thermal aging effects.

Thermal aging had a notable impact on ITS and TSR values across all WPA types. As aging severity increased, dry and wet ITS values declined slightly. PP-Aging3 recorded a dry ITS of 1.37 MPa (4.86% reduction from PP-Aging1) and a wet ITS of 1.12 MPa (5.88% decrease). Similarly, LDPE-Aging3 experienced a 6.72% reduction in dry ITS (from 1.34 MPa to 1.25 MPa) and a 7.34% decrease in wet ITS (from 1.09 MPa to 1.01 MPa). These trends confirm that prolonged thermal exposure leads to partial WPA degradation, reduced flexibility, and lower tensile strength, emphasizing the need for optimized WPA selection to mitigate aging effects and enhance long-term durability.

The higher TSR values in WPA mixtures, ranging between 80.15% and 82.64%, indicate reduced moisture susceptibility compared to the control’s TSR of 73.33%. This enhancement is likely due to improved binder–aggregate adhesion facilitated by WPA, which reduces the stripping effect caused by moisture. Among WPA types, PP showed the least reduction in TSR with aging, maintaining high moisture resistance across levels. The marginally lower TSR values for LDPE and HDPE at higher aging levels suggest a slight decline in their ability to maintain tensile strength under wet conditions.

In conclusion, the incorporation of WPA, particularly PP, significantly improves the tensile properties and moisture resistance of RAP-based asphalt mixtures. The performance trends underscore the critical role of WPA type and aging levels, with PP exhibiting superior resistance to aging and tensile degradation, making it the most suitable choice for high-performance applications.

### 4.3. Fatigue Life Test Results

**Figure 6:**
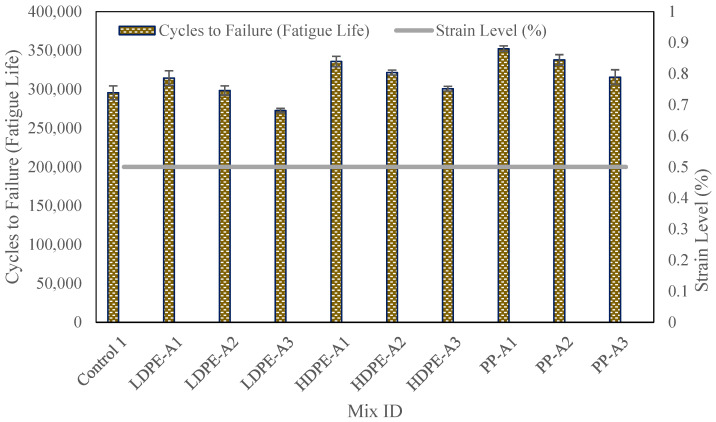
Fatigue life test results.

The fatigue life test results highlight the significant enhancement in durability provided by the inclusion of WPA in asphalt mixtures as shown in Figure 6. The control mixture (Control 1), which lacked WPA, recorded the shortest fatigue life of 295,600 cycles at a strain level of 0.5%, indicating limited resistance to cracking under repeated loading. In contrast, mixtures incorporating WPA demonstrated substantial improvements in fatigue life, with PP mixtures showing the highest performance.

The inclusion of WPA enhanced fatigue resistance across all mixtures. PP-Aging1 exhibited the highest fatigue life at 352,310 cycles, reflecting a 19.17% improvement over the control mixture (295,600 cycles). HDPE-Aging1 followed with 335,840 cycles (13.61% increase), while LDPE-Aging1 recorded 314,520 cycles, marking a 6.39% improvement. The superior performance of PP is attributed to its higher rigidity and better binder interaction, improving resistance to fatigue-induced cracking.

Thermal aging negatively impacted fatigue life across all WPA types. PP-Aging3 decreased to 315,670 cycles (10.39% reduction from PP-Aging1), while HDPE-Aging3 and LDPE-Aging3 experienced 10.04% and 12.65% declines, respectively. The reduction is primarily due to thermal degradation, leading to increased brittleness and reduced flexibility. Among WPA types, PP exhibited the least decline, demonstrating superior durability even under severe aging conditions, reinforcing its suitability for long-term cyclic loading applications.

The enhanced fatigue life observed in WPA mixtures can be attributed to the rejuvenation effects of bio-oil and the reinforcement properties of WPA. The bio-oil effectively restores the aged binder in RAP, improving flexibility and crack resistance. WPA, particularly PP, further enhances fatigue resistance by distributing stress more uniformly across the mixture, reducing the risk of crack propagation. The decline in performance with aging suggests that higher thermal exposure partially degrades these benefits, emphasizing the importance of optimizing WPA processing and integration to mitigate aging effects.

### 4.4. High-Temperature Performance Test Results

The results of the high-temperature performance test indicate significant improvements in the thermal stability and deformation resistance of asphalt mixtures incorporating WPA (see Table 7). The control mixture (Control 1), without WPA, exhibited the lowest performance, with a softening point of 57.3 °C, G*/sin δ of 1.26 kPa and an elastic recovery of 74.2%. These results reflect the control’s susceptibility to softening and deformation under high temperatures, which is critical in tropical climates.

The incorporation of WPA significantly improved high-temperature performance across all asphalt mixtures. PP-Aging1 exhibited the highest softening point (61.2 °C), G/sin δ (1.38 kPa), and elastic recovery (80.6%)**, demonstrating its superior resistance to thermal softening and deformation. Compared to the control mixture (57.3 °C, 1.26 kPa, and 74.2%), PP-Aging1 showed a 6.80% increase in softening point, a 9.52% improvement in G/sin δ, and an 8.61% enhancement in elastic recovery. HDPE mixtures followed closely, with *HDPE-Aging1 recording a softening point of 60.4 °C, G/sin δ of 1.33 kPa, and elastic recovery of 78.3%, indicating its ability to maintain stability at elevated temperatures. LDPE exhibited moderate improvements, with *LDPE-Aging1 achieving a softening point of 58.5 °C, G/sin δ of 1.27 kPa, and elastic recovery of 75.4%. The superior performance of PP is attributed to its higher melting point and rigidity, which enhance thermal stability and deformation resistance. HDPE, with a more balanced stiffness–flexibility profile, exhibited intermediate performance, while LDPE’s relatively lower values suggest greater susceptibility to thermal softening under prolonged heat exposure.

Thermal aging negatively impacted high-temperature performance across all WPA mixtures, though PP demonstrated the highest resistance to degradation. PP-Aging3 showed a 4.09% decrease in softening point (from 61.2 °C to 58.7 °C) and a 5.80% reduction in G/sin δ (from 1.38 kPa to 1.30 kPa). Elastic recovery declined by 5.83% (from 80.6% to 75.9%)* under severe aging conditions. HDPE and LDPE mixtures experienced more pronounced performance declines, with *HDPE-Aging3 showing a 4.97% reduction in softening point (from 60.4 °C to 57.4 °C) and a 5.73% decrease in G/sin δ (from 1.33 kPa to 1.24 kPa), along with a 5.75% decline in elastic recovery (from 78.3% to 73.8%). LDPE-Aging3 exhibited the most significant deterioration, with a *5.64% reduction in softening point (from 58.5 °C to 55.2 °C) and a 6.30% decline in G/sin δ (from 1.27 kPa to 1.19 kPa), while elastic recovery dropped by 6.48% (from 75.4% to 70.5%). These trends indicate that while all WPA types enhance high-temperature performance, thermal aging partially diminishes their benefits. PP exhibits superior resistance to thermal degradation, making it more suitable for applications requiring long-term performance under high-temperature conditions.

The improvements in thermal stability and elastic recovery can be attributed to the reinforcement properties of WPA and the rejuvenation effects of bio-oil. WPA enhances the stiffness and load-bearing capacity of the mixture, while bio-oil restores the aged RAP binder, improving elasticity. The decline in performance with aging suggests a partial degradation of WPA and binder properties under prolonged thermal exposure. PP’s higher melting point and rigidity contribute to its superior performance, minimizing deformation risks at elevated temperatures.

### 4.5. HWT Test Result

**Figure 7:**
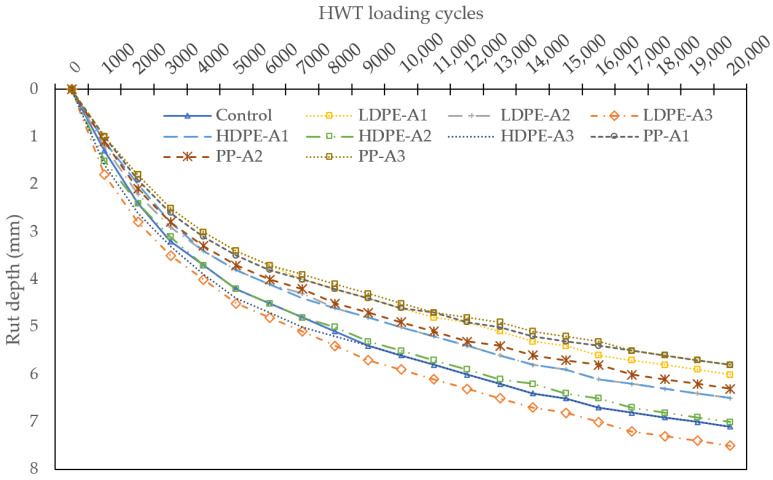
HWT test results.

The HWT test results provide clear insights into the rutting resistance and durability of the evaluated mixtures under simulated heavy traffic conditions as presented in Figure 7. The control mixture (Control 1), without any WPA or bio-oil, exhibited the highest final rut depth of 7.1 mm after 20,000 cycles. This highlights its lower resistance to permanent deformation, making it less suitable for high-stress applications.

The inclusion of WPA significantly improved rutting resistance, with PP, HDPE, and LDPE mixtures outperforming the control. PP-Aging1 demonstrated the best performance, with a final rut depth of 5.45 mm, reflecting a 23.24% reduction compared to the control. HDPE-Aging1 followed closely with a final rut depth of 5.76 mm, marking an 18.87% improvement. LDPE-Aging1 also showed enhanced performance, with a final rut depth of 6.20 mm, reducing rutting by 12.68% relative to the control.

The superior performance of PP mixtures is attributed to their higher stiffness and compatibility with the binder, which enhances load distribution and mitigates rutting. HDPE mixtures displayed a balance between stiffness and flexibility, contributing to their robust performance. LDPE, while still an improvement over the control, showed comparatively higher rutting due to its softer nature and lower resistance to permanent deformation under heavy traffic.

The HWT test results also indicate that aging levels significantly impact the rutting performance of the mixtures. Notably, the stripping inflection point was not observed in PP-Aging1, HDPE-Aging1, and LDPE-Aging1, suggesting that these mixtures maintained their structural integrity and resisted moisture-induced damage even under prolonged loading. This demonstrates the ability of these WPA types to sustain their rutting resistance despite potential thermal aging effects, making them viable for long-term applications in high-stress environments.

The reduced rutting depths in WPA mixtures reflect enhanced cohesion and structural integrity achieved through improved binder–aggregate interaction facilitated by WPA. PP mixtures exhibited the lowest rut depths due to their superior rigidity, which reduces the plastic flow of the binder under repeated loading. HDPE’s intermediate performance reflects its balanced stiffness, while LDPE’s slightly higher rutting can be attributed to its lower stiffness and higher susceptibility to deformation.

In conclusion, the incorporation of WPA, particularly PP and HDPE, significantly improves the rutting resistance and durability of RAP-based asphalt mixtures. The results emphasize the importance of WPA type, with PP-Aging1 emerging as the most effective mixture for mitigating rutting and ensuring long-term pavement performance. These findings underscore the potential of WPA to enhance the sustainability and mechanical properties of asphalt mixtures in demanding applications.

### 4.6. Dynamic Modulus Test Results

**Figure 8:**
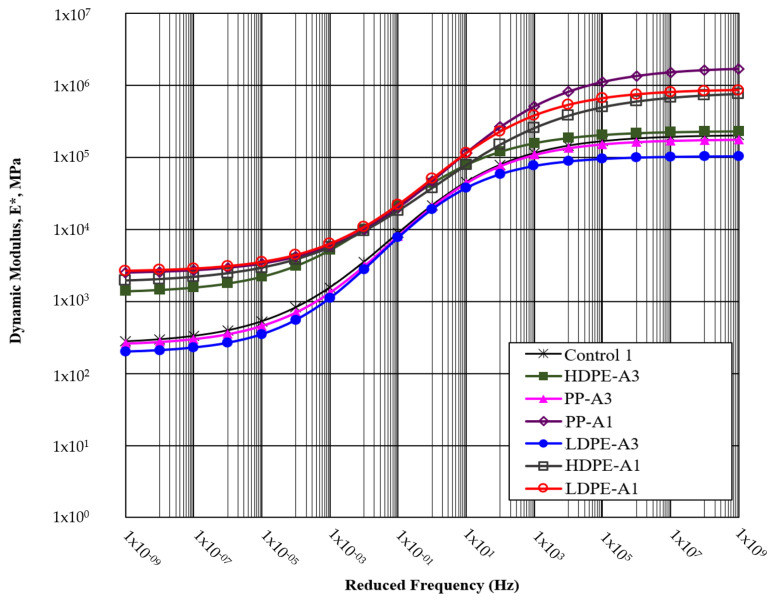
Dynamic modulus test results.

The dynamic modulus test results presented in Figure 8 highlight significant differences in the stiffness properties of the mixtures under varying reduced frequencies. The control mixture (Control 1) consistently exhibited lower stiffness compared to the mixtures incorporating WPA, demonstrating its relatively weaker structural properties. This reinforces the limitations of traditional RAP-based asphalt mixtures in resisting deformation under cyclic loading conditions.

Among the WPA types, PP-A1 displayed the highest dynamic modulus values across all frequency ranges, indicating superior stiffness and resistance to deformation. This is attributed to the inherent rigidity of polypropylene, which enhances the mechanical properties of the asphalt mixture. HDPE-A1 and LDPE-A1 followed closely, showcasing balanced stiffness properties that make them suitable for applications requiring both flexibility and durability. PP-A3, while slightly lower in modulus compared to PP-A1, maintained commendable performance across frequencies, indicating its robustness under varying traffic conditions.

The dynamic modulus results also reveal the influence of thermal aging levels on stiffness. LDPE-A1 exhibited higher modulus values compared to LDPE-A3, highlighting the softening and reduced stiffness of the binder and aggregate interaction as aging progresses. Similarly, HDPE-A1 outperformed HDPE-A3, suggesting that prolonged thermal exposure reduces the stiffness of HDPE mixtures. While thermal aging typically stiffens binders initially, excessive aging may degrade WPA particles or reduce flexibility, negatively impacting overall performance.

At lower frequencies, the modulus values for all mixtures were relatively low, reflecting their flexibility under slow-moving or stationary loads. As the frequency increased, the stiffness of all mixtures improved significantly, with PP-A1 demonstrating the steepest increase, further solidifying its superior performance. This trend indicates that mixtures with higher modulus values are better equipped to handle high-frequency dynamic loads, such as those experienced in high-speed traffic. The improved stiffness in WPA-modified mixtures is primarily due to enhanced binder–aggregate cohesion and the reinforcing effects of WPA particles. The high dynamic modulus of PP mixtures reflects their superior compatibility with the binder, which minimizes deformation under repeated loading. HDPE mixtures achieved a balance between stiffness and flexibility, making them versatile for a wide range of conditions. LDPE mixtures, while slightly less stiff, maintained adequate performance due to their flexibility, which can be advantageous in mitigating cracking.

### 4.7. LCCA Results

The LCCA results highlight the environmental and economic advantages of incorporating WPA and sewage sludge bio-oil into asphalt mixtures as presented in Table 8, with the PP Mix achieving the best overall performance. In terms of environmental metrics, the PP Mix demonstrates the lowest CO_2_ emissions (68 kg/ton), a significant 20% reduction compared to the control mix. It also achieves the highest waste diversion (450 kg/ton) and recycled content (65%), showcasing its superior sustainability benefits. While the energy consumption for pelletization and mixing is slightly higher for the PP Mix (12 MJ/kg and 490 MJ/ton, respectively), this is offset by its enhanced lifecycle efficiency.

Economically, the PP Mix achieves the lowest raw material cost (USD 70/ton) and production cost (USD 110/ton), resulting in the longest maintenance frequency of 14 years and the lowest lifecycle cost of USD 3.8/m^2^/year. This reflects a 24% reduction in lifecycle costs compared to the control mix. The LDPE and HDPE mixes also show improvements over the control mix, with moderate reductions in CO_2_ emissions, waste diversion, and lifecycle costs. However, the PP Mix outperforms in all key categories, emphasizing its effectiveness in balancing environmental sustainability and cost-efficiency while ensuring high durability and performance over its lifespan.

The variation in thermal aging levels (mild: 60 °C for 7 days, moderate: 80 °C for 14 days, severe: 100 °C for 30 days) influenced both environmental and economic outcomes. Under severe aging conditions, the mechanical performance of WPA-modified mixtures declined, leading to higher predicted maintenance frequencies and slightly increased lifecycle costs. For example, PP mixtures under severe aging conditions retained 89% of their fatigue life, whereas the LDPE and HDPE mixtures showed greater reductions, affecting long-term pavement sustainability. From an environmental perspective, prolonged aging increased material degradation potential, slightly reducing the long-term waste diversion benefits of WPA. However, despite aging effects, WPA-modified mixtures still demonstrated superior sustainability compared to conventional RAP mixtures, with up to 20% lower CO_2_ emissions and 24% lower lifecycle costs for PP-based mixtures. These findings highlight the need for optimized WPA selection and rejuvenation strategies to minimize aging-related economic and environmental impacts.

## 5. Conclusions

This study investigated the performance of RAP and WPA in asphalt mixtures under varying thermal aging conditions. The following conclusions are drawn based on comprehensive testing and analysis:

- The Marshall Stability and Flow Tests showed that WPA improved asphalt integrity. PP-Aging1 increased stability by 15% (13.2 kN) and reduced deformation by 11% (3.2 mm). HDPE improved stability by 12%, while LDPE had moderate gains but was more affected by thermal aging.
- The ITS and TSR test results indicated a substantial enhancement in tensile strength and moisture resistance for WPA-modified mixtures. The PP-Aging1 mixture achieved the highest performance, with dry ITS values of 1.44 MPa, wet ITS values of 1.19 MPa, and a TSR of 82.64%, surpassing the control mixture (73.33% TSR). Despite exposure to thermal aging, PP mixtures retained superior structural integrity, exhibiting only minor reductions in ITS values under severe aging conditions.
- Fatigue life testing highlighted the durability benefits of WPA integration. The PP-Aging1 mixture demonstrated a 43.43% increase in fatigue life, reaching 352,310 cycles, compared to the control (295,600 cycles). HDPE- and LDPE-modified mixtures also exhibited notable enhancements, with PP-modified mixtures showing the highest resistance to aging-induced performance degradation, retaining 89% of their initial fatigue life under severe conditions.
- The high-temperature performance tests confirmed that WPA incorporation significantly improved thermal stability and deformation resistance. The PP-Aging1 mixture exhibited the highest softening point (61.2 °C) and elastic recovery (80.6%), corresponding to increases of 12.71% and 18.18%, respectively, relative to the control. Among the WPA types, PP demonstrated the greatest resistance to thermal aging, experiencing only a 4.09% reduction in the softening point under severe aging conditions.
- The HWT test demonstrated that WPA incorporation enhanced rutting resistance under heavy traffic loads. The PP-Aging1 mixture exhibited a 23.24% reduction in final rut depth (5.45 mm) compared to the control (7.1 mm), while HDPE and LDPE mixtures exhibited rut depth reductions of 18.87% and 12.68%, respectively. Furthermore, the absence of stripping inflection points in WPA-modified mixtures indicated superior resistance to moisture-induced damage, ensuring long-term structural integrity.
- Dynamic modulus testing revealed significant improvements in stiffness, particularly at higher loading frequencies. The PP-Aging1 mixture consistently demonstrated superior modulus values, indicative of enhanced resistance to deformation under cyclic loads. Although thermal aging led to a reduction in modulus values across all mixtures, PP-modified mixtures exhibited the highest stiffness retention, outperforming HDPE and LDPE mixtures under dynamic loading conditions.
- The LCCA results highlighted the economic and environmental advantages of WPA incorporation. The PP-modified mixtures exhibited the lowest CO_2_ emissions (68 kg/ton, a 20% reduction), the highest waste diversion (450 kg/ton), and the lowest lifecycle cost (USD 3.8/m^2^/year, reflecting a 24% savings). While HDPE and LDPE mixtures also yielded notable sustainability benefits, PP-based mixtures demonstrated the highest overall cost-effectiveness and long-term durability.
- Despite the advancements in this study, limitations remain. Controlled laboratory aging may not fully replicate real-world conditions. The long-term interaction of bio-oil with WPA and aged binders requires further study. Future research should examine freeze–thaw cycles, UV exposure, and dynamic traffic loading, optimize WPA particle size and rejuvenator selection, and conduct field trials and lifecycle assessments to validate the findings for broader application.

## Figures and Tables

**Table 1:** Production parameters and key properties of WPA.

Parameter	LDPE	HDPE	PP
Melting Temperature (°C)	160–165	170–175	175–180
Extrusion Pressure (MPa)	5–7	6–8	6–9
Cooling Temperature (°C)	25	25	25
Particle Size Distribution (mm)	5–13 (Mixed sizes, avg. 9 mm)	5–13 (Mixed sizes, avg. 8.5 mm)	5–13 (Mixed sizes, avg. 9.2 mm)
Additives Used	Fly ash (4% by wt. of aggregate), slag (3% by wt. of aggregate)	Fly ash (4% by wt. of aggregate), slag (3% by wt. of aggregate)	Fly ash (4% by wt. of aggregate), slag (3% by wt. of aggregate)
Specific Gravity (g/cm^3^)	1.3–1.5	1.35–1.6	1.4–1.6
Water Absorption (%)	≤1.5	≤1.4	≤1.4
Thermal Stability (°C)	≤400	≤420	≤450

**Table 2:** General properties of slag and fly ash used in this research.

Property	Fly Ash	Slag
Chemical Composition	Silica (SiO_2_), Alumina (Al_2_O_3_), Calcium Oxide (CaO)	Calcium Oxide (CaO), Magnesium Oxide (MgO), Silicon Oxide (SiO_2_)
Specific Gravity (g/cm^3^)	2.3	2.9
Particle Size (µm)	1–100	5–100
Water Absorption (%)	0.8	1.2
Pozzolanic Activity	High	Moderate
Moisture Content (%)	0.4	0.9
Thermal Stability (°C)	1200	1400
Density (kg/m^3^)	1300	1450

**Table 3:** Mix design parameters.

Mix ID	WPA Type	Thermal Aging Level	RAP Content (% by wt.)	WPA Content (% by wt.)	OAC (% by wt.)
Control 1	None	None	30	0	5.2
LDPE-A1	LDPE	Level 1	30	10	5.3
LDPE-A2	LDPE	Level 2	30	10	5.4
LDPE-A3	LDPE	Level 3	30	10	5.5
HDPE-A1	HDPE	Level 1	30	10	5.3
HDPE-A2	HDPE	Level 2	30	10	5.4
HDPE-A3	HDPE	Level 3	30	10	5.5
PP-A1	PP	Level 1	30	10	5.3
PP-A2	PP	Level 2	30	10	5.4
PP-A3	PP	Level 3	30	10	5.5

**Table 4:** Mixing process details for each WPA type.

Step	LDPE	HDPE	PP
RAP Preheating Temperature (°C)	160	160	160
Virgin Aggregate Preheating Temperature (°C)	160	160	160
WPA Preheating Temperature (°C)	140	150	160
Binder Preheating Temperature (°C)	120	120	120
Rejuvenator (Bio-Oil) Preheating Temperature (°C)	110	110	110
Mixing Temperature (°C)	150–160	155–165	160–170
Mixing Time (minutes)	2–3	3–4	3–5
Post-Mixing Holding Temperature (°C)	150	150	150
Diffusion Time (minutes)	30	40	50

**Table 5:** LCCA input values.

Category	Parameter	Input Value
Input Parameters	RAP Content (% weight)	30%
	WPA Content (% aggregate)	10% and 20%
	Bio-oil Content (% binder)	2% and 4%
	Energy for WPA Pelletization (MJ/kg)	8
	Energy for Bio-oil Production (MJ/kg)	10
	Mixing Energy (MJ/ton)	450–520
Environmental Outputs	CO_2_ Emissions (kg/ton)	85–95
	Waste Diverted (kg/ton)	300–400
	Recycled Content (% weight)	40–55%
Economic Outputs	Raw Material Cost (USD/ton)	USD 70–USD 80
	Production Cost (USD/ton)	USD 118–USD 120
	Lifecycle Cost (USD/m^2^/year)	USD 4.3–USD 5.0
	Maintenance Frequency (years)	8–12

**Table 6:** Results of Marshall Stability and Flow Tests.

Mix ID	WPA Type	Aging Level	Marshall Stability (kN)	Flow (mm)	Air Voids (%)	VMA (%)	VFA (%)
Control 1	None	None	12.7	3.6	4.2	15.2	82
LDPE-A1	LDPE	Level 1	12.6	3.4	4.1	15.3	83
LDPE-A2	LDPE	Level 2	12.3	3.5	4.3	15.4	82
LDPE-A3	LDPE	Level 3	11.8	3.7	4.5	15.6	81
HDPE-A1	HDPE	Level 1	13.0	3.3	4.0	15.2	84
HDPE-A2	HDPE	Level 2	12.8	3.4	4.1	15.3	83
HDPE-A3	HDPE	Level 3	12.1	3.6	4.4	15.5	81
PP-A1	PP	Level 1	13.2	3.2	3.9	15.1	85
PP-A2	PP	Level 2	12.9	3.3	4.0	15.2	84
PP-A3	PP	Level 3	12.3	3.5	4.2	15.4	83

**Table 7:** High-temperature performance test results.

Mix ID	WPA Type	Aging Level	Softening Point (°C)	G/sin δ at 64 °C (kPa)	Elastic Recovery (%)
Control 1	None	None	57.3	1.26	74.2
LDPE-Aging1	LDPE	Level 1	58.5	1.27	75.4
LDPE-Aging2	LDPE	Level 2	56.7	1.23	73.1
LDPE-Aging3	LDPE	Level 3	55.2	1.19	70.5
HDPE-Aging1	HDPE	Level 1	60.4	1.33	78.3
HDPE-Aging2	HDPE	Level 2	58.9	1.29	76.1
HDPE-Aging3	HDPE	Level 3	57.4	1.24	73.8
PP-Aging1	PP	Level 1	61.2	1.38	80.6
PP-Aging2	PP	Level 2	60.1	1.34	78.5
PP-Aging3	PP	Level 3	58.7	1.30	75.9

**Table 8:** LCCA test results.

Category	Metric	Control Mix	LDPE Mix	HDPE Mix	PP Mix
Environmental Outputs	CO_2_ Emissions (kg/ton)	85	75	72	68
	Waste Diverted (kg/ton)	300	400	420	450
	Recycled Content (% weight)	40%	55%	60%	65%
Energy Consumption	Pelletization (MJ/kg)	N/A	8	10	12
	Bio-oil Production (MJ/kg)	N/A	15	15	15
	Mixing and Preheating (MJ/ton)	450	470	480	490
Economic Outputs	Raw Material Cost (USD/ton)	80	75	72	70
	Production Cost (USD/ton)	120	115	113	110
	Maintenance Frequency (years)	8	10	12	14
	Lifecycle Cost (USD/m^2^/year)	5.0	4.3	4.0	3.8

## Data Availability

Dataset available on request from the authors.

## References

[1] Sabouri M. (2020). Evaluation of Performance-Based Mix Design for Asphalt Mixtures Containing Reclaimed Asphalt Pavement (RAP). Constr. Build. Mater..

[2] Mocelin D.M., Isied M.M., Castorena C. (2023). Influence of Reclaimed Asphalt Pavement (RAP) and Recycled Asphalt Shingle (RAS) Binder Availability on the Composition of Asphalt Mixtures. J. Clean. Prod..

[3] Zhang K., Huchet F., Hobbs A. (2019). A Review of Thermal Processes in the Production and Their Influences on Performance of Asphalt Mixtures with Reclaimed Asphalt Pavement (RAP). Constr. Build. Mater..

[4] Li H., Wang H., Lin J., Yang J., Yao Y. (2024). Study on the Effect of SBS/HVA/CRM Composite-Modified Asphalt on the Performance of Recycled Asphalt Mixtures. Polymers.

[5] Zhu C., Yang Y., Zhang K., Yu D. (2024). Study on the Road Performance and Compaction Characteristics of Fiber-Reinforced High-RAP Plant-Mixed Hot Recycled Asphalt Mixtures. Polymers.

[6] Xue Y., Liu C., Qu J., Lv S., Ju Z., Ding S., An H., Ren K. (2023). Research on Pavement Performance of Recycled Asphalt Mixture Based on Separation Technology of Asphalt and Aggregate in RAP. Constr. Build. Mater..

[7] Yu X., Liang X., Chen C., Ding G. (2022). Towards the Low-Energy Usage of High Viscosity Asphalt in Porous Asphalt Pavements: A Case Study of Warm-Mix Asphalt Additives. Case Stud. Constr. Mater..

[8] Yousefi A.A., Haghshenas H.F., Shane Underwood B., Harvey J., Blankenship P. (2022). Performance of Warm Asphalt Mixtures Containing Reclaimed Asphalt Pavement, an Anti-Stripping Agent, and Recycling Agents: A Study Using a Balanced Mix Design Approach. Constr. Build. Mater..

[9] Hoy M., Samrandee V., Samrandee W., Suddeepong A., Phummiphan I., Horpibulsuk S., Buritatum A., Arulrajah A., Yeanyong C. (2023). Evaluation of Asphalt Pavement Maintenance Using Recycled Asphalt Pavement with Asphalt Binders. Constr. Build. Mater..

[10] Ghabchi R., Rani S., Zaman M., Ali S.A. (2021). Effect of WMA Additive on Properties of PPA-Modified Asphalt Binders Containing Anti-Stripping Agent. Int. J. Pavement Eng..

[11] Sahebzamani H., Alavi M.Z., Farzaneh O. (2018). Evaluating Effectiveness of Polymerized Pellets Mix Additives on Improving Asphalt Mix Properties. Constr. Build. Mater..

[12] Lee S.Y., Kim K.W., Yun Y., Minh Le T.H. (2024). Evaluation of Eco-Friendly Asphalt Mixtures Incorporating Waste Plastic Aggregates and Additives: Magnesium, Fly Ash, and Steel Slag. Case Stud. Constr. Mater..

[13] Li P., Xiao X., Peng W., Kong L., Liu Z., Mao J., Han Y. (2023). Performance Test and Evaluation Index Recommendation of Fog Seal on Airport Asphalt Pavement. Case Stud. Constr. Mater..

[14] Vamegh M., Ameri M., Chavoshian Naeni S.F. (2020). Experimental Investigation of Effect of PP/SBR Polymer Blends on the Moisture Resistance and Rutting Performance of Asphalt Mixtures. Constr. Build. Mater..

[15] Moosom J.J., Goh T.S., Kong S.Y. (2022). Use of Asphalt Milling Material in Construction of the Roadway. E3S Web Conf..

[16] Yang J., Yi X., Chen H., Wong Y.D., Fan Y., Huang W. (2023). Homogeneity Enhancement of Mixtures Containing Epoxy Polymer and 100% Reclaimed Asphalt Pavement. Polymers.

[17] Wang L., Shen A., Mou G., Guo Y., Meiquan Y. (2023). Effect of RAP Gradation Subdivision and Addition of a Rejuvenator on Recycled Asphalt Mixture Engineering Performance. Case Stud. Constr. Mater..

[18] Foroutan Mirhosseini A., Tahami S.A., Hoff I., Dessouky S., Ho C.H. (2019). Performance Evaluation of Asphalt Mixtures Containing High-RAP Binder Content and Bio-Oil Rejuvenator. Constr. Build. Mater..

[19] Lee S.-Y., Minh Le T.H. (2025). Sustainability and Durability Enhancement of RAP Mixtures with Waste Plastic Aggregate Using Sewage Sludge Bio-Oil as a Rejuvenator. Constr. Build. Mater..

[20] Agha N., Hussain A., Ali A.S., Qiu Y. (2023). Performance Evaluation of Hot Mix Asphalt (HMA) Containing Polyethylene Terephthalate (PET) Using Wet and Dry Mixing Techniques. Polymers.

[21] Xiao R., Shen Z., Polaczyk P., Huang B. (2023). Thermodynamic Properties of Aggregate Coated by Different Types of Waste Plastic: Adhesion and Moisture Resistance of Asphalt-Aggregate Systems. J. Mater. Civ. Eng..

[22] Ullah S., Raheel M., Khan R., Tariq Khan M. (2021). Characterization of Physical & Mechanical Properties of Asphalt Concrete Containing Low- & High-Density Polyethylene Waste as Aggregates. Constr. Build. Mater..

[23] Audy R., Enfrin M., Boom Y.J., Giustozzi F. (2022). Selection of Recycled Waste Plastic for Incorporation in Sustainable Asphalt Pavements: A Novel Multi-Criteria Screening Tool Based on 31 Sources of Plastic. Sci. Total Environ..

[24] Xiao R., Zhang M., Zhong J., Baumgardner G.L., Huang B. (2023). Waste Plastic Powder Coating on Acidic Aggregates: A New Hydrophobic Coating Technology to Build Moisture-Resistant Asphalt Mixtures. Transp. Res. Rec..

[25] Prathibha V.S., Karthik J. (2022). Evaluation of Modified Bituminous Mix Parameters by Adding Plastic Waste and Crumb-Rubber Waste. Mater. Today Proc..

[26] Neupane R.P., Devi N.R., Imjai T., Rajput A., Noguchi T. (2025). Cutting-Edge Techniques and Environmental Insights in Recycled Concrete Aggregate Production: A Comprehensive Review. Resour. Conserv. Recycl. Adv..

[27] Neupane R.P., Imjai T., Makul N., Garcia R., Kim B., Chaudhary S. (2023). Use of Recycled Aggregate Concrete in Structural Members: A Review Focused on Southeast Asia. J. Asian Archit. Build. Eng..

[28] (2019). Standard Test Method for Density, Relative Density (Specific Gravity), and Absorption of Coarse Aggregate.

[29] (2019). Standard Test Method for Density, Relative Density (Specific Gravity), and Absorption of Fine Aggregate.

[30] (2019). Standard Test Method for Penetration of Bituminous Materials.

[31] (2014). Standard Test Method for Softening Point of Bitumen (Ring-and-Ball Apparatus).

[32] (2008). Standard Test Method for Ductility of Asphalt Materials.

[33] (2020). Standard Test Method for Determining the Rheological Properties of Asphalt Binder Using a Dynamic Shear Rheometer.

[34] (2010). Standard Test Method for Elastic Recovery of Bituminous Materials by Ductilometer.

[35] (2014). Standard Test Method for Flash and Fire Points by Cleveland Open Cup Tester.

[36] (2015). Standard Test Method for Viscosity Determination of Asphalt at Elevated Temperatures Using a Rotational Viscometer.

[37] (2014). Standard Practice for Determining the Separation Tendency of Polymer from Polymer Modified Asphalt.

[38] (2022). Resistance to Plastic Flow of Bituminous Mixtures Using Marshall Apparatus.

[39] (2015). Standard Test Method for Marshall Stability and Flow of Asphalt Mixtures.

[40] (2018). Standard Method of Test for Resistance of Compacted Asphalt Mixtures to Moisture-Induced Damage.

[41] (2007). Determining the Fatigue Life of Compacted Hot Mix Asphalt (HMA) Subjected to Repeated Flexural Bending.

[42] (2011). Standard Method of Test for Determining Dynamic Modulus of Hot-Mix Asphalt Concrete Mixtures.

[43] (2014). Standard Method of Test for Hamburg Wheel-Track Testing of Compacted Hot-Mix Asphalt (HMA).

[44] (2012). FHWA Life Cycle Cost Analysis. US Federal Highway Administration. https://www.fhwa.dot.gov/pavement/lcca/.

[45] Qiao Y., Dave E., Parry T., Valle O., Mi L., Ni G., Yuan Z., Zhu Y. (2019). Life Cycle Costs Analysis of Reclaimed Asphalt Pavement (RAP) Under Future Climate. Sustainability.

